# *N*-Cyclo­hexyl-*N*′-(4-nitro­benzo­yl)thio­urea

**DOI:** 10.1107/S1600536810012249

**Published:** 2010-04-10

**Authors:** Sohail Saeed, Naghmana Rashid, Wing-Tak Wong

**Affiliations:** a Department of Chemistry, Research Complex, Allama Iqbal Open University, Islamabad, Pakistan; b Department of Applied Biology and Chemical Technology, The Hong Kong Polytechnic University, Hung Hom, Kowloon, Hong Kong SAR, People’s Republic of China

## Abstract

In the title compound, C_14_H_17_N_3_O_3_S, the nitro group is twisted slightly by 2.6 (3)° from the benzene ring plane and the thio­ureido group makes a dihedral angle of 52.06 (4)° with the benzene ring. The cyclo­hexyl ring displays a chair conformation. An intra­molecular N—H⋯O inter­action is present. In the crystal, inter­molecular N—H⋯S hydrogen bonds link the mol­ecules into centrosymmetric dimers. π–π inter­actions between inversion-related benzene rings (centroid–centroid distance = 4.044 Å) and C—H⋯π inter­actions (H⋯centroid distance = 3.116 Å) between one methyl­ene cyclo­hexyl H atom and the benzene ring are also present.

## Related literature

For general background to the chemistry and biological activity of thio­urea derivatives and their use as organic synthons or as complexing agents, see: Glasser & Doughty (1964 ▶); Jain & Rao (2003 ▶); Zeng *et al.* (2003 ▶); Xu *et al.* (2004 ▶); Zheng *et al.* (2004 ▶); D’hooghe *et al.* (2005 ▶); Saeed *et al.* (2008 ▶, 2009 ▶, 2010 ▶). 
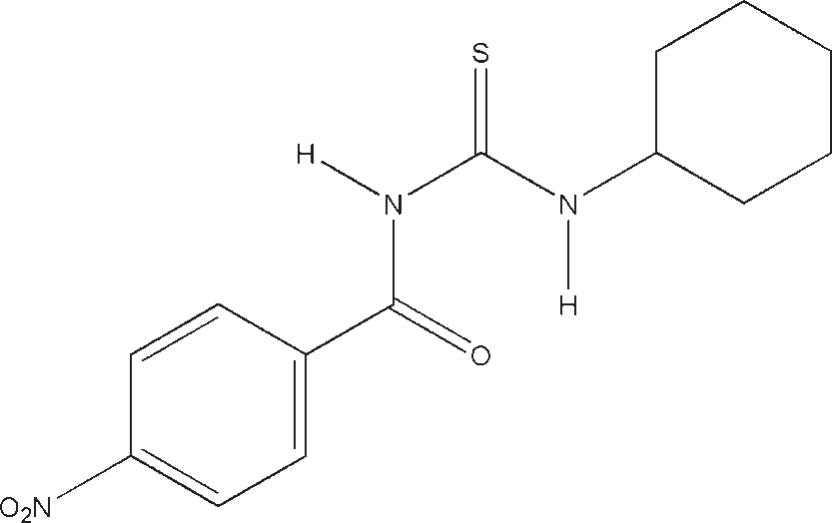


## Experimental

### 

#### Crystal data

C_14_H_17_N_3_O_3_S
*M* *_r_* = 307.37
Monoclinic, 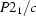

*a* = 10.7865 (7) Å
*b* = 6.9218 (4) Å
*c* = 20.6788 (13) Å
β = 101.493 (1)°
*V* = 1512.96 (16) Å^3^
*Z* = 4
Mo *K*α radiation
μ = 0.23 mm^−1^
*T* = 294 K
0.43 × 0.32 × 0.26 mm

#### Data collection

Bruker SMART 1000 CCD diffractometer
Absorption correction: multi-scan (*SADABS*; Bruker, 2001 ▶) *T*_min_ = 0.909, *T*_max_ = 0.943
10042 measured reflections
3683 independent reflections
3177 reflections with *I* > 2σ(*I*)
*R*_int_ = 0.017

#### Refinement

*R*[*F* ^2^ > 2σ(*F* ^2^)] = 0.037
*wR*(*F* ^2^) = 0.108
*S* = 1.04
3683 reflections
200 parameters
H atoms treated by a mixture of independent and constrained refinement
Δρ_max_ = 0.25 e Å^−3^
Δρ_min_ = −0.20 e Å^−3^

### 

Data collection: *SMART* (Bruker, 2006 ▶); cell refinement: *SAINT* (Bruker, 2006 ▶); data reduction: *SAINT*; program(s) used to solve structure: *SHELXS97* (Sheldrick, 2008 ▶); program(s) used to refine structure: *SHELXL97* (Sheldrick, 2008 ▶); molecular graphics: *ORTEPII* (Johnson, 1976 ▶); software used to prepare material for publication: *SHELXL97*.

## Supplementary Material

Crystal structure: contains datablocks global, I. DOI: 10.1107/S1600536810012249/bh2277sup1.cif

Structure factors: contains datablocks I. DOI: 10.1107/S1600536810012249/bh2277Isup2.hkl

Additional supplementary materials:  crystallographic information; 3D view; checkCIF report

## Figures and Tables

**Table 1:** Hydrogen-bond geometry (Å, °)

*D*—H⋯*A*	*D*—H	H⋯*A*	*D*⋯*A*	*D*—H⋯*A*
N3—H3*N*⋯O3	0.817 (17)	1.981 (17)	2.6507 (17)	138.8 (14)
N2—H2*N*⋯S1^i^	0.84 (2)	2.67 (2)	3.4999 (12)	171.3 (16)

Symmetry code: (i) 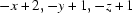.

**Fig. 1.**
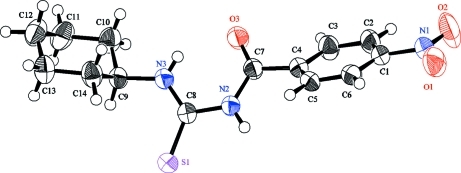
ORTEP plot of the title compound, with 30% probability thermal ellipsoids and the atom numbering scheme.

**Fig. 2.**
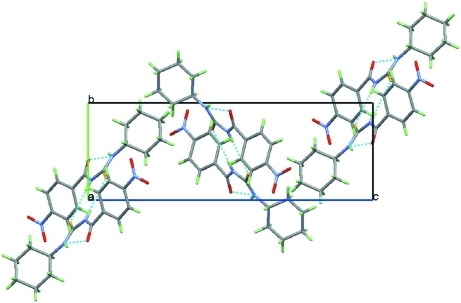
The unit cell packing diagram of the title compound, viewed down the a-axis. The cyan dotted lines represent the inter-and intra-molecular H-bonding interactions.

## supplementary crystallographic information

### Comment

Thiourea and its derivatives have found extensive applications in the fields of medicine, agriculture and analytical chemistry. Substituted thioureas are an important class of compounds, precursors or intermediates towards the synthesis of a variety of heterocyclic systems such as imidazole-2-thiones (Zeng *et al.*, 2003), 2-imino-1,3-thiazolines (D'hooghe *et al.*, 2005) pyrimidines-2-thione (Jain & Rao, 2003) and (benzothiazolyl)-4-quinazolinones. *N*-(Substituted phenyl)-*N*-phenylthioureas and *N*-(substituted butanoyl)-*N*-phenylthioureas have been developed. Thioureas are also known to exhibit a wide range of biological activities including antiviral, antibacterial, antifungal, anticancer (Saeed *et al.*, 2010) antitubercular, antithyroidal, herbicidal and insecticidal activities and as agrochemicals (Xu *et al.*, 2004), *e.g*. 1-benzoyl-3-(4,5-disubstituted-pyrimidine-2-yl)-thioureas, which have excellent herbicidal activity (Zheng *et al.*, 2004). Thioureas are also well known chelating agents for transition metals (Saeed *et al.*, 2009). *N*,*N*-Dialkyl-*N*'-benzoyl thioureas act as selective complexing agents for the enrichment of platinum metals even from strongly interfacing matrixes. The complexes of thiourea derivatives also show various biological activities (Glasser & Doughty, 1964). Thiourea derivatives containing the amino functional groups are also used as epoxy crosslinking agents (Saeed *et al.*, 2008, 2009).

The title compound, *N*-cyclohexyl-*N*'-(4-nitrobenzoyl)-thiourea, crystallizes in a monoclinic primitive space group, *P*2_1_/*c* (#14). Like other analogues, the molecule is not planar. The nitro group, N1/O1/O2, is slightly twisted [2.6 (3)°] from the benzene ring plane (C1···C6). For the he thioureido group, the mean plane defined by C7/O3/N2/C8/S1/N3 is twisted by 52.06 (4)° from the benzene ring plane. The cyclohexyl ring is in the chair form.

Most of the bond lengths in the molecule are within 0.01 Å of the mean and median of comparable bond types in the CSD database.

There are intra-molecular N—H···O H-bond interactions. The intermolecular N—H···S H-bond interactions link the molecules to form dimers in the crystal lattice. There are also π···π interactions between neighbouring benzene rings and C13—H13B···π interactions between the cyclohexyl H atom and the benzene ring in the crystal lattice. The distance between the atom H13B and the centroid of C1···C6 benzene ring is 3.116 Å. The centroid-to-centroid distance of the ring C1···C6 and (C1···C6)* (* symmetry code: 1-*x*, 1-*y*, 1-z) is 4.044 Å and the distance between C5* and centroid of C1···C6 is 3.610 Å.

### Experimental

A solution of 4-nitrobenzoyl chloride (0.01 mol) in dry acetone (80 ml) was added dropwise to a suspension of ammonium thiocyanate (0.01 mol) in acetone (50 ml) and the reaction mixture was refluxed for 45 minutes. After cooling to room temperature, a solution of cyclohexyl amine (0.01 mol) in acetone (25 ml) was added and the resulting mixture refluxed for 2 h. The reaction mixture was poured into five times its volume of cold water, upon which the thiourea precipitated. The product was recrystallized from ethyl acetate as yellow block crystals.

### Refinement

Although all C-bound H atoms may be found in a difference map, they were placed in geometrical idealized positions, with C—H bond lengths fixed to 0.93, 0.97 and 0.98 Å for phenyl, methylene and methine H atoms, respectively. All C-bound H-atoms were refined using a riding model, with *U*_iso_(H) = 1.2*U*_eq_(carrier C atom). Atoms H2N and H3N, bonded to N2 and N3, were located in a difference map and refined isotropically with free coordinates.

### Crystal data

**Table:**

C_14_H_17_N_3_O_3_S	*F*(000) = 648
*M**_r_* = 307.37	*D*_x_ = 1.349 Mg m^−^^3^
Monoclinic, *P*2_1_/*c*	Melting point: 389 K
Hall symbol: -P 2ybc	Mo *K*α radiation, λ = 0.71073 Å
*a* = 10.7865 (7) Å	Cell parameters from 10042 reflections
*b* = 6.9218 (4) Å	θ = 1.9–28.3°
*c* = 20.6788 (13) Å	µ = 0.23 mm^−^^1^
β = 101.493 (1)°	*T* = 294 K
*V* = 1512.96 (16) Å^3^	Block, yellow
*Z* = 4	0.43 × 0.32 × 0.26 mm

### Data collection

**Table:**

Bruker SMART 1000 CCD diffractometer	3683 independent reflections
Radiation source: fine-focus sealed tube	3177 reflections with *I* > 2σ(*I*)
graphite	*R*_int_ = 0.017
ω scans	θ_max_ = 28.3°, θ_min_ = 1.9°
Absorption correction: multi-scan (*SADABS*; Bruker, 2001)	*h* = −14→14
*T*_min_ = 0.909, *T*_max_ = 0.943	*k* = −9→5
10042 measured reflections	*l* = −27→21

### Refinement

**Table:**

Refinement on *F*^2^	Secondary atom site location: difference Fourier map
Least-squares matrix: full	Hydrogen site location: inferred from neighbouring sites
*R*[*F*^2^ > 2σ(*F*^2^)] = 0.037	H atoms treated by a mixture of independent and constrained refinement
*wR*(*F*^2^) = 0.108	*w* = 1/[σ^2^(*F*_o_^2^) + (0.057*P*)^2^ + 0.3039*P*] where *P* = (*F*_o_^2^ + 2*F*_c_^2^)/3
*S* = 1.04	(Δ/σ)_max_ = 0.002
3683 reflections	Δρ_max_ = 0.25 e Å^−^^3^
200 parameters	Δρ_min_ = −0.20 e Å^−^^3^
0 restraints	Extinction correction: *SAINT* (Bruker, 2006), Fc^*^=kFc[1+0.001xFc^2^λ^3^/sin(2θ)]^-1/4^
0 constraints	Extinction coefficient: 0.0071 (14)
Primary atom site location: structure-invariant direct methods	

### Fractional atomic coordinates and isotropic or equivalent isotropic displacement parameters (Å^2^)

**Table:**

	*x*	*y*	*z*	*U*_iso_*/*U*_eq_	
S1	1.03975 (3)	0.73010 (6)	0.436999 (18)	0.05209 (13)	
O1	0.47704 (13)	0.08216 (19)	0.65091 (7)	0.0781 (4)	
O2	0.44185 (17)	0.3242 (2)	0.70892 (8)	0.1000 (5)	
O3	0.67743 (10)	0.91616 (15)	0.49838 (6)	0.0595 (3)	
N1	0.48457 (12)	0.2530 (2)	0.66423 (7)	0.0597 (3)	
N2	0.83598 (10)	0.70331 (17)	0.49038 (5)	0.0439 (2)	
N3	0.83313 (11)	0.94898 (16)	0.41468 (6)	0.0474 (3)	
C1	0.55199 (11)	0.3817 (2)	0.62577 (6)	0.0469 (3)	
C2	0.56593 (15)	0.5738 (2)	0.64356 (7)	0.0554 (3)	
H2	0.5347	0.6211	0.6792	0.066*	
C3	0.62757 (15)	0.6938 (2)	0.60694 (7)	0.0543 (3)	
H3	0.6368	0.8244	0.6174	0.065*	
C4	0.67605 (11)	0.61980 (19)	0.55443 (6)	0.0429 (3)	
C5	0.66178 (11)	0.42564 (19)	0.53834 (6)	0.0437 (3)	
H5	0.6951	0.3766	0.5035	0.052*	
C6	0.59808 (12)	0.30409 (19)	0.57394 (7)	0.0461 (3)	
H6	0.5868	0.1740	0.5631	0.055*	
C7	0.72985 (13)	0.76095 (18)	0.51229 (6)	0.0440 (3)	
C8	0.89672 (11)	0.80319 (18)	0.44630 (6)	0.0404 (3)	
C9	0.87497 (12)	1.07460 (18)	0.36608 (6)	0.0450 (3)	
H9	0.9669	1.0915	0.3785	0.054*	
C10	0.81166 (18)	1.2697 (2)	0.36788 (8)	0.0581 (4)	
H10A	0.8380	1.3263	0.4114	0.070*	
H10B	0.7206	1.2526	0.3598	0.070*	
C11	0.84568 (19)	1.4059 (2)	0.31634 (8)	0.0675 (4)	
H11A	0.7997	1.5262	0.3168	0.081*	
H11B	0.9354	1.4348	0.3275	0.081*	
C12	0.81434 (18)	1.3191 (3)	0.24797 (8)	0.0660 (4)	
H12A	0.7234	1.3052	0.2346	0.079*	
H12B	0.8429	1.4054	0.2170	0.079*	
C13	0.87599 (18)	1.1253 (3)	0.24606 (8)	0.0650 (4)	
H13A	0.9671	1.1419	0.2539	0.078*	
H13B	0.8492	1.0695	0.2025	0.078*	
C14	0.84263 (16)	0.9873 (2)	0.29742 (7)	0.0567 (4)	
H14A	0.7529	0.9581	0.2866	0.068*	
H14B	0.8888	0.8673	0.2967	0.068*	
H2N	0.8739 (16)	0.605 (3)	0.5076 (8)	0.056 (4)*	
H3N	0.7671 (16)	0.977 (2)	0.4261 (8)	0.054 (4)*	

### Atomic displacement parameters (Å^2^)

**Table:**

	*U*^11^	*U*^22^	*U*^33^	*U*^12^	*U*^13^	*U*^23^
S1	0.04215 (19)	0.0602 (2)	0.0569 (2)	0.00659 (13)	0.01688 (14)	0.01451 (15)
O1	0.0819 (8)	0.0661 (8)	0.0935 (9)	−0.0121 (6)	0.0350 (7)	0.0161 (7)
O2	0.1244 (13)	0.0977 (10)	0.1022 (10)	−0.0046 (9)	0.0809 (10)	0.0102 (8)
O3	0.0656 (6)	0.0483 (5)	0.0731 (7)	0.0133 (5)	0.0342 (5)	0.0136 (5)
N1	0.0497 (7)	0.0726 (9)	0.0615 (7)	0.0012 (6)	0.0222 (6)	0.0175 (6)
N2	0.0459 (5)	0.0424 (6)	0.0467 (6)	0.0050 (4)	0.0169 (4)	0.0081 (4)
N3	0.0476 (6)	0.0455 (6)	0.0535 (6)	0.0053 (5)	0.0207 (5)	0.0107 (5)
C1	0.0406 (6)	0.0552 (7)	0.0471 (6)	0.0039 (5)	0.0143 (5)	0.0114 (6)
C2	0.0654 (8)	0.0587 (8)	0.0488 (7)	0.0079 (7)	0.0275 (6)	0.0022 (6)
C3	0.0688 (9)	0.0463 (7)	0.0536 (7)	0.0041 (6)	0.0261 (7)	−0.0004 (6)
C4	0.0429 (6)	0.0454 (6)	0.0426 (6)	0.0039 (5)	0.0139 (5)	0.0048 (5)
C5	0.0419 (6)	0.0474 (7)	0.0448 (6)	0.0050 (5)	0.0161 (5)	0.0005 (5)
C6	0.0424 (6)	0.0444 (6)	0.0531 (7)	0.0024 (5)	0.0136 (5)	0.0039 (5)
C7	0.0475 (6)	0.0434 (6)	0.0439 (6)	0.0018 (5)	0.0159 (5)	0.0026 (5)
C8	0.0424 (6)	0.0409 (6)	0.0391 (6)	−0.0020 (5)	0.0105 (5)	0.0002 (5)
C9	0.0463 (6)	0.0417 (6)	0.0494 (7)	−0.0014 (5)	0.0154 (5)	0.0079 (5)
C10	0.0789 (10)	0.0421 (7)	0.0566 (8)	0.0046 (6)	0.0211 (7)	0.0018 (6)
C11	0.0914 (12)	0.0421 (7)	0.0679 (10)	−0.0033 (8)	0.0134 (8)	0.0105 (7)
C12	0.0729 (10)	0.0653 (10)	0.0565 (8)	−0.0053 (8)	0.0048 (7)	0.0172 (7)
C13	0.0809 (10)	0.0681 (10)	0.0510 (8)	−0.0059 (8)	0.0253 (7)	0.0041 (7)
C14	0.0738 (9)	0.0459 (7)	0.0563 (8)	−0.0025 (6)	0.0269 (7)	−0.0018 (6)

### Geometric parameters (Å, °)

**Table:**

S1—C8	1.6707 (13)	C5—H5	0.9300
O1—N1	1.2132 (19)	C6—H6	0.9300
O2—N1	1.2162 (19)	C9—C10	1.5173 (19)
O3—C7	1.2213 (15)	C9—C14	1.518 (2)
N1—C1	1.4785 (17)	C9—H9	0.9800
N2—C7	1.3717 (16)	C10—C11	1.521 (2)
N2—C8	1.4058 (16)	C10—H10A	0.9700
N2—H2N	0.835 (18)	C10—H10B	0.9700
N3—C8	1.3182 (16)	C11—C12	1.511 (2)
N3—C9	1.4665 (15)	C11—H11A	0.9700
N3—H3N	0.817 (17)	C11—H11B	0.9700
C1—C6	1.3774 (18)	C12—C13	1.501 (3)
C1—C2	1.379 (2)	C12—H12A	0.9700
C2—C3	1.380 (2)	C12—H12B	0.9700
C2—H2	0.9300	C13—C14	1.524 (2)
C3—C4	1.3932 (17)	C13—H13A	0.9700
C3—H3	0.9300	C13—H13B	0.9700
C4—C5	1.3857 (18)	C14—H14A	0.9700
C4—C7	1.5008 (17)	C14—H14B	0.9700
C5—C6	1.3869 (17)		
			
O1—N1—O2	123.31 (14)	C10—C9—C14	110.89 (12)
O1—N1—C1	118.89 (13)	N3—C9—H9	108.9
O2—N1—C1	117.78 (15)	C10—C9—H9	108.9
C7—N2—C8	126.77 (11)	C14—C9—H9	108.9
C7—N2—H2N	117.8 (12)	C9—C10—C11	111.20 (13)
C8—N2—H2N	115.1 (12)	C9—C10—H10A	109.4
C8—N3—C9	126.38 (11)	C11—C10—H10A	109.4
C8—N3—H3N	115.9 (11)	C9—C10—H10B	109.4
C9—N3—H3N	117.4 (11)	C11—C10—H10B	109.4
C6—C1—C2	123.12 (12)	H10A—C10—H10B	108.0
C6—C1—N1	118.42 (13)	C12—C11—C10	111.65 (13)
C2—C1—N1	118.46 (12)	C12—C11—H11A	109.3
C1—C2—C3	118.19 (12)	C10—C11—H11A	109.3
C1—C2—H2	120.9	C12—C11—H11B	109.3
C3—C2—H2	120.9	C10—C11—H11B	109.3
C2—C3—C4	120.20 (13)	H11A—C11—H11B	108.0
C2—C3—H3	119.9	C13—C12—C11	111.23 (14)
C4—C3—H3	119.9	C13—C12—H12A	109.4
C5—C4—C3	120.17 (12)	C11—C12—H12A	109.4
C5—C4—C7	121.95 (11)	C13—C12—H12B	109.4
C3—C4—C7	117.52 (12)	C11—C12—H12B	109.4
C4—C5—C6	120.26 (12)	H12A—C12—H12B	108.0
C4—C5—H5	119.9	C12—C13—C14	111.98 (13)
C6—C5—H5	119.9	C12—C13—H13A	109.2
C1—C6—C5	118.04 (12)	C14—C13—H13A	109.2
C1—C6—H6	121.0	C12—C13—H13B	109.2
C5—C6—H6	121.0	C14—C13—H13B	109.2
O3—C7—N2	123.77 (12)	H13A—C13—H13B	107.9
O3—C7—C4	119.66 (11)	C9—C14—C13	111.10 (12)
N2—C7—C4	116.56 (11)	C9—C14—H14A	109.4
N3—C8—N2	115.75 (11)	C13—C14—H14A	109.4
N3—C8—S1	125.16 (10)	C9—C14—H14B	109.4
N2—C8—S1	119.09 (9)	C13—C14—H14B	109.4
N3—C9—C10	108.03 (11)	H14A—C14—H14B	108.0
N3—C9—C14	111.11 (11)		

### Hydrogen-bond geometry (Å, °)

**Table:**

*D*—H···*A*	*D*—H	H···*A*	*D*···*A*	*D*—H···*A*
N3—H3N···O3	0.817 (17)	1.981 (17)	2.6507 (17)	138.8 (14)
N2—H2N···S1^i^	0.84 (2)	2.67 (2)	3.4999 (12)	171.3 (16)
C6—H6···O3^ii^	0.93	2.54	3.3041 (17)	140
C9—H9···S1	0.98	2.82	3.1555 (13)	101

Symmetry codes: (i) −*x*+2, −*y*+1, −*z*+1; (ii) *x*, *y*−1, *z*.
